# Impact of Antiretroviral Therapy on Incidence of Pregnancy among HIV-Infected Women in Sub-Saharan Africa: A Cohort Study

**DOI:** 10.1371/journal.pmed.1000229

**Published:** 2010-02-09

**Authors:** Landon Myer, Rosalind J. Carter, Monica Katyal, Patricia Toro, Wafaa M. El-Sadr, Elaine J. Abrams

**Affiliations:** 1 Centre for Infectious Diseases Epidemiology & Research, School of Public Health & Family Medicine, University of Cape Town, Cape Town, South Africa; 2 International Center for AIDS Care and Treatment Programs, Columbia University Mailman School of Public Health, New York, United States of America; National Institute of Child Health and Human Development, United States of America

## Abstract

**Background:**

With the rapid expansion of antiretroviral therapy (ART) services in sub-Saharan Africa there is growing recognition of the importance of fertility and childbearing among HIV-infected women. However there are few data on whether ART initiation influences pregnancy rates.

**Methods and Findings:**

We analyzed data from the Mother-to-Child Transmission-Plus (MTCT-Plus) Initiative, a multicountry HIV care and treatment program for women, children, and families. From 11 programs in seven African countries, women were enrolled into care regardless of HIV disease stage and followed at regular intervals; ART was initiated according to national guidelines on the basis of immunological and/or clinical criteria. Standardized forms were used to collect sociodemographic and clinical data, including incident pregnancies. Overall 589 incident pregnancies were observed among the 4,531 women included in this analysis (pregnancy incidence, 7.8/100 person-years [PY]). The rate of new pregnancies was significantly higher among women receiving ART (9.0/100 PY) compared to women not on ART (6.5/100 PY) (adjusted hazard ratio, 1.74; 95% confidence interval, 1.19–2.54). Other factors independently associated with increased risk of incident pregnancy included younger age, lower educational attainment, being married or cohabiting, having a male partner enrolled into the program, failure to use nonbarrier contraception, and higher CD4 cell counts.

**Conclusions:**

ART use is associated with significantly higher pregnancy rates among HIV-infected women in sub-Saharan Africa. While the possible behavioral or biomedical mechanisms that may underlie this association require further investigation, these data highlight the importance of pregnancy planning and management as a critical but neglected component of HIV care and treatment services.

*Please see later in the article for the Editors' Summary*

## Abstract 2

A multicountry cohort study in sub-Saharan Africa by Landon Myer and colleagues reveals higher pregnancy rates in HIV-infected women on antiretroviral therapy (ART).

## Editors' Summary

**Background:**

Human immunodeficiency virus (HIV) causes Acquired Immunodeficiency Syndrome (AIDS), which is a major global cause of disease and death. More than 33 million people around the world are infected with HIV, with nearly 5,500 dying daily from HIV and AIDS-related complications. HIV/AIDS is especially problematic in sub-Saharan Africa, where it is the leading cause of death. There is no cure for HIV/AIDS, but medicines known as “antiretroviral therapy” (ART) can prolong life and reduce complications in patients infected with HIV. 97% of patients with HIV/AIDS live in low- and middle-income countries. According to the World Health Organization, nearly 10 million of these patients need ART. As patients' access to treatment is often hindered by the high cost and low availability of ART, global health efforts have focused on promoting ART use in resource-limited nations. Such efforts also increase awareness of how HIV is spread (contact with blood or semen, in sexual intercourse, sharing needles, or from mother to child during childbirth). ART reduces, but does not remove, the chance of a mother's passing HIV to her child during birth.

**Why Was This Study Done?:**

By the end of 2007, 3 million HIV-infected patients in poor countries were receiving ART. Many of those treated with ART are young women of child-bearing age. Childbirth is an important means of spreading HIV in sub-Saharan Africa, where 60% of all HIV patients are women. This study questions whether the improved health and life expectancy that results from treatment with ART affects pregnancy rates of HIV-infected patients. The study explores this question in seven African countries, by examining the rates of pregnancy in HIV-infected women before and after they started ART.

**What Did the Researchers Do and Find?:**

The authors looked at the records of 4,531 HIV-infected women enrolled in the Mother-to-Child-Transmission-Plus (MTCT-Plus) Initiative in seven African countries. MTCT -Plus, begun in 2002, is a family-centered treatment program that offers regular checkups, blood tests, counseling, and ART treatment (if appropriate) to women and their families. At each checkup, women's CD4+ cell counts and World Health Organization guidelines were used to determine their eligibility for starting ART. Over a 4-year period, nearly a third of the women starting ART experienced a pregnancy: 244 pregnancies occurred in the “pre-ART” group (women not receiving ART) compared to 345 pregnancies in the “on-ART” group (women receiving ART). The chance of pregnancy increased over time in the on-ART group to almost 80% greater than the pre-ART group, while remaining relatively low and constant in the pre-ART group. The authors noted that, as expected, other factors also increased the chances of pregnancy, including younger age, lower educational status, and use of nonbarrier contraception such as injectable hormones.

**What Do These Findings Mean?:**

This study suggests that starting ART is associated with higher pregnancy rates in sub-Saharan Africa, nearly doubling the chances of a woman becoming pregnant. The reasons for this link are unclear. One possible explanation is behavioral: women receiving ART may feel more motivated to have children as their health and quality of life improve. However, the study did not examine how pregnancy desires and sexual activity of women changed while on ART, and cannot discern why ART is linked to increased pregnancy. By using pregnancy data gathered from patient questionnaires rather than laboratory tests, the study is limited by the possibility of inaccurate patient reporting. Understanding how pregnancy rates vary in HIV-infected women receiving ART helps support the formation of responsive, effective HIV programs. Female HIV patients of child-bearing age, who form the majority of patients receiving ART in sub-Saharan Africa, would benefit from programs that combine starting HIV treatment with ART with education and contraception counseling and pregnancy-related care.

**Additional Information:**

Please access these Web sites via the online version of this summary at http://dx.doi.org/10.1371/journal.pmed.1000229.

- Information is available from the US National Institute of Allergy and Infectious Diseases on HIV infection and AIDS
- HIV InSite has comprehensive information on all aspects of HIV/AIDS, including a list of articles and other sources of information about the primary care of adolescents with HIV
- A UNAIDS 2008 report is available on the global AIDS epidemic
- The International Planned Parenthood Foundation provides information on sexual and reproductive health and HIV
- The International Center for AIDS Care and Treatment Programs at the Columbia University Mailman School of Public health provides information to assist HIV care and treatment programs in resource-limited settings

## Introduction

By the end of 2007 there were almost 3,000,000 HIV-infected individuals receiving antiretroviral therapy (ART) in resource-limited settings [1]. The global roll-out of ART has contributed to a greater awareness of issues related to fertility and childbearing among HIV-infected women and men [2],[3], particularly in sub-Saharan Africa where a large proportion of HIV-infected individuals are women in their reproductive years and the prevention of mother-to-child transmission (PMTCT) of HIV is an ongoing challenge [1],[4].

Studies from Europe and North America indicate that HIV-infected women frequently become pregnant [5],[6], and most HIV-infected individuals have fertility desires that change over time [7]. Data are sparse from Africa however, where socioeconomic and cultural imperatives have a substantial impact on female fertility [8]. Small qualitative studies from Africa suggest that HIV might modify but does not eliminate broader desires to have children [9],[10] and that ART use may be associated with increased fertility desires among HIV-infected women, possibly through increased hopes and planning for the future [11].

HIV care and treatment services are in a unique position to address the childbearing desires of HIV-infected individuals as well as to ensure safe pregnancy and delivery [12]. These services have the opportunity to prevent unwanted pregnancies through provision of effective contraception [13], taking into account potential interactions between antiretroviral drugs and hormonal contraceptives. HIV care and treatment services can also optimize ART for women before conception and during pregnancy to decrease the risk of vertical transmission of HIV and to avoid the use of potentially teratogenic drugs [4] thus ensuring optimal outcomes in these women and their children [14],[15].

Despite the importance of fertility and childbearing in HIV-infected individuals, little is known about whether initiation of ART alters pregnancy rates among women in HIV care and treatment programs in resource-limited settings. One study from the United States suggested that ART use did not impact pregnancy rates [5], but there are no data from sub-Saharan Africa. We examined the incidence of pregnancy before and after ART initiation in women enrolled in a multicountry HIV care and treatment program in Africa.

## Methods

### The MTCT-Plus Initiative

Data for this analysis come from the MTCT-Plus Initiative, a multicountry HIV care and treatment program that utilizes a woman-centered, family-focused service model. The design and progress of the Initiative has been previously described (www.mtctplus.org) [16],[17]. At each site, pregnant or recently postpartum HIV-infected women receiving PMTCT services were enrolled into HIV care and treatment services irrespective of HIV disease stage (“index women”). Infected children, partners, and other family members were also offered enrollment. Services were initiated during 2003 and 2004.

### HIV Care and Treatment Services

All enrolled women received a package of HIV primary care services including regular clinical assessments and CD4 cell counts every 6 mo. Barrier and nonbarrier contraceptive methods were provided, either on-site or by referral, with specific methods based on local availability. There was no standardized counseling on pregnancy and contraceptive use across sites, although all sites provided counseling and psychosocial support services regarding HIV-related stigma, disclosure, and treatment adherence.

ART initiation was based on national or World Health Organization (WHO) guidelines [18]. Typically this entailed ART initiation for patients with WHO stage 4 disease, CD4 cell counts ≤200 cells/µl, or WHO stage 3 and CD4 cell counts ≤350 cells/µl. First-line regimens involved two nucleoside reverse transcriptase inhibitors and a non-nucleoside reverse transcriptase inhibitor (primarily nevirapine). The follow-up schedule at each site was quarterly to semi-annually for individuals not receiving ART (“pre-ART”) and monthly for individuals receiving ART (“on-ART”).

### Measurements

Upon enrollment women completed a short, standardized provider-administered questionnaire, which included demographic and socioeconomic information, partnership status, and obstetric history. At enrollment and each follow-up visit, clinicians completed a standardized medical survey including history of opportunistic infections, WHO stage, initiation or continuing use of ART, contraceptive use (including the use of specific barrier and nonbarrier methods), and pregnancy status (including pregnancy loss since the previous visit). The gestational age of pregnancies at detection was estimated clinically (on the basis of dates of last menstrual period or symphysis-fundal palpation), and estimated delivery dates were calculated accordingly. CD4 cell enumeration was conducted at local laboratories.

### Ethical Approval

The conduct of the MTCT-Plus Initiative as a service delivery program with data collection for monitoring and evaluation purposes was approved by the Institutional Review Board of Columbia University.

### Data Analysis

Data were analyzed using Stata version 10.0 (Stata Corporation). Timing of incident pregnancies was based on estimated conception dates, calculated 280 d before the date of delivery (estimated or observed). Pregnancy-free survival was calculated from 60 d following the delivery of the index pregnancy (during or after which women were enrolled into the program) until either: incident pregnancy, death, loss to follow-up, or censoring at the last clinical visit recorded before 31 January 2007. Participants were censored after their first new pregnancy during follow-up. Women who reported being sterilized or having a hysterectomy were excluded. Follow-up of women prior to initiation of ART was designated as the “pre-ART period,” while follow-up after initiation of ART is referred to as the “on-ART” period.

Median values were compared using the rank-sum test and proportions were compared using chi-square tests replaced by Fisher's exact test for sparse data. Pregnancy rates were calculated with 95% confidence intervals (CIs), and crude rates were compared as incident pregnancy rate ratios (RRs). The time to pregnancy was compared between subgroups using proportional hazards models with the Breslow method for tie handling. The results of these models are expressed as hazard ratios (HRs) with 95% CI. In these models, the clustering of women within sites was accounted for using the sandwich-Huber-White robust variance estimation method [19]. Parameters of interest that varied during the course of follow-up (including ART use, contraceptive use, current CD4 cell count, and enrollment of a male partner into the program) were analyzed as time-dependent covariates. On the basis of the final model we plotted the pregnancy-free survival function for women during the pre-ART and on-ART periods, adjusted for relevant covariates. To assess the changing rate of pregnancy over time for women during the pre-ART and on-ART periods, the instantaneous hazard of pregnancy from proportional hazards models was graphed with corresponding 95% CI using a smoothing function. All statistical tests are two-sided at α = 0.05.

## Results

### Baseline Characteristics

A total of 4,531 women enrolled at 11 sites between February 2003 and January 2007 were eligible for analysis. The median number of women enrolled per site was 410 (range, 211–666). Table 1 describes the cohort overall and by age group. The median age was 27 y (interquartile range (IQR), 24–31 y). The median parity was 2 (including the index pregnancy; IQR, 1–3); this increased with age, and overall 22% of women were primiparous during follow-up. The median CD4 cell count at enrollment was 366 cells/µl (IQR, 208–562). At the first follow-up visit, 39% of women (*n* = 1,755) reported contraceptive use, including barrier methods (17% reported condom use, *n* = 752), injectable hormonal methods (15%, *n* = 664), oral hormonal contraceptives (4%, *n* = 160), intrauterine device (IUD) use (1%, *n* = 31), or other methods (3%, *n* = 148). 259 women (6%) reported using condoms as well as a nonbarrier method.

**Table 1:** Description of sociodemographic and clinical characteristics of the cohort of women enrolled into MTCT-Plus and eligible for incident pregnancy analysis, overall and by age category.

Characteristics	All Participants 4,531 (100%)	Age Category			
		<25 y, *n* = 1,373 (30%)	25–29 y, *n* = 1,574 (35%)	30–34 y, *n* = 1,094 (24%)	35+ y, *n* = 484 (11%)
**Median years of schooling (y)**	9	9	9	9	8
**Years (y) of education**					
0–3	602 (13)	170 (12)	199 (13)	153 (14)	80 (17)
4–7	1,170 (26)	348 (25)	369 (23)	297 (27)	156 (33)
8–11	1,644 (36)	572 (42)	553 (35)	362 (33)	157 (32)
12+	1,109 (25)	283 (21)	453 (29)	282 (26)	91 (19)
**Employed**	1,045 (23)	195 (14)	387 (25)	315 (29)	148 (31)
**Household electricity**	2,721 (60)	726 (54)	1,028 (65)	680 (63)	278 (58)
**Household piped water**	1,919 (43)	535 (39)	715 (46)	466 (43)	203 (42)
**Currently married/cohabiting**	2,929 (66)	821 (61)	1,044 (67)	741 (68)	323 (70)
**Male partner enrolled into the program**	1,010 (22)	306 (22)	368 (23)	246 (22)	90 (19)
**Parity: median**	2	2	2	3	4
**Primiparous**	1,013 (22)	613 (45)	307 (20)	88 (8)	11 (2)
**Estimated travel time to facility (min)**					
<30	1,623 (36)	511 (37)	565 (36)	375 (34)	172 (36)
30–60	2,169 (48)	680 (50)	736 (47)	544 (50)	209 (43)
60+	725 (16)	179 (13)	271 (17)	172 (16)	103 (21)
**WHO stage at enrollment**					
1	2,911 (64)	966 (70)	1012 (64)	641 (59)	292 (60)
2	911 (20)	262 (19)	320 (20)	241 (22)	88 (18)
3	624 (14)	127 (9)	212 (13)	196 (18)	89 (18)
4	76 (2)	17 (1)	29 (2)	15 (1)	15 (3)
**Median CD4 at enrollment (cells/µl)**	366	442	353	322	319
≥500	1,709 (38)	649 (47)	568 (36)	328 (30)	158 (33)
350–499	863 (19)	278 (20)	295 (19)	207 (19)	83 (17)
20–349	984 (22)	256 (19)	360 (23)	260 (24)	108 (22)
<200	975 (22)	190 (14)	351 (22)	299 (27)	135 (28)
**Initiated ART during follow-up**	1,978 (44)	461 (34)	712 (45)	550 (50)	255 (53)

Approximately half of the women (56%, *n* = 2,551) were observed only during the pre-ART period; 580 women (13%) were observed only on-ART, and another 1,400 (31%) were observed both pre-ART and on-ART.

### Incidence of Pregnancy during Follow-up

The median duration of follow-up was 482 d during pre-ART and 696 d on-ART. Overall, there were 7,565 person-years of observation following the index pregnancy and 589 incident pregnancies were detected (incidence rate, 7.79 per 100 person-years; 95% CI 7.18–8.43). This total was comprised of 244 incident pregnancies over 3,747 person-years of observation during the pre-ART period (rate, 6.51 per 100 person-years; 95% CI 5.73–7.38) and 345 incident pregnancies over 3,817 person-years of observation during the on-ART period (rate, 9.03 per 100 person-years; 95% CI 8.13–10.03).

Crude rates of incident pregnancy varied by site and country, with rates ranging from 3.29 pregnancies per 100 person-years in South Africa (95% CI 2.60–4.18) to 21.68 per 100 person-years in Rwanda (95% CI 17.10–27.50); the incidence of pregnancy was between 6 and 9 per 100 person-years in each of the remaining countries.

**Figure 1:**
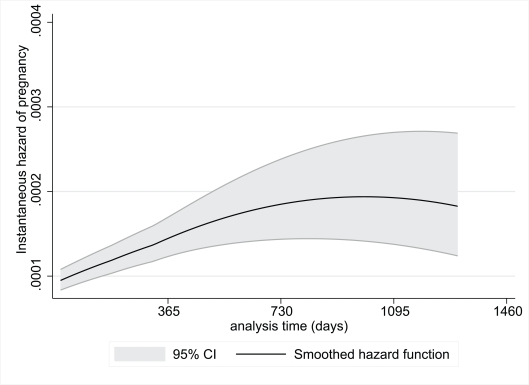
Instantaneous hazard of pregnancy during the pre-ART period by duration of follow-up, with 95% CIs.

**Figure 2:**
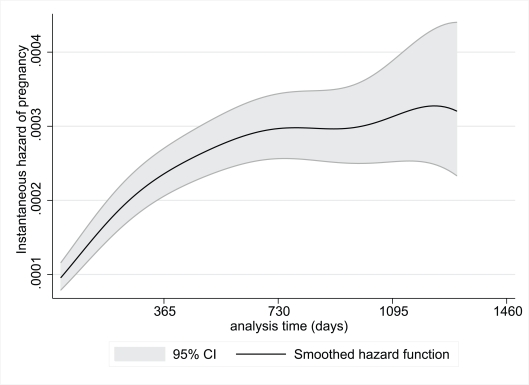
Instantaneous hazard of pregnancy during the on-ART period by duration of follow-up, with 95% CIs.

Figures 1 and 2 show the risk of pregnancy over time during the pre-ART and on-ART periods, respectively. During the pre-ART period the risk of pregnancy was lower and relatively constant, peaking before 3 y of follow-up. In contrast, the risk of pregnancy appeared to increase continuously with increasing duration of follow-up in women on-ART, with pregnancy rates on-ART of 6.8, 9.9, 10.5, and 13.7 pregnancies per 100 woman-years of observation during each of the first 4 y after ART initiation, respectively (*p*<0.001).

### Factors Associated with Incident Pregnancies

Table 2 shows the crude incidence of pregnancy during the pre-ART and on-ART periods by women's demographic and clinical characteristics. Rates of pregnancy decreased sharply with increasing age in all time periods; combining both the pre-ART and on-ART periods, women <25 y of age had more than double the rate of new pregnancies compared to women ≥35 y. Higher socioeconomic status (as reflected by education, employment, access to electricity, and piped water) was associated with lower rates of pregnancy, although the associations did not achieve statistical significance in all intervals. Women who were married or cohabiting at enrollment, and women who had male partners enrolled into the program, also had significantly increased rates of pregnancy. Primiparous women had similar pregnancy rates compared to multiparous women and there was not a trend towards lower pregnancy rates with increased parity. More advanced WHO stage at enrollment was significantly associated with decreased pregnancy rate during the pre-ART period but not for women on-ART.

**Table 2:** Crude incidence rates of pregnancy per 100 person-years of observation, with 95% CIs, among women according to selected participant demographic, socioeconomic, clinical, and immunological characteristics.

Characteristics	Incidence Rate (95% CI)		
	Overall	Pre-ART	On-ART
**Overall**	7.78 (7.18–8.43)	6.51 (5.74–7.38)	9.03 (8.13–10.04)
***Baseline characteristics***			
**Age (y)**			
<25	9.69 (8.43–11.12)	8.51 (7.04–10.29)	11.45 (9.36–14.00)
25–29	8.46 (7.42–9.63)	6.04 (4.84–7.53)	10.75 (9.15–12.63)
30–34	6.47 (5.44–7.70)	5.62 (4.25–7.44)	7.14 (5.72–8.91)
35+	4.06 (2.90–5.69) (*p*<0.001)	3.15 (1.70–5.86) (*p* = 0.001)	4.62 (3.10–6.90) (*p*<0.001)
**Years of education (y)**			
0–3	13.75 (10.16–18.60)	13.60 (8.68–21.32)	13.86 (9.21–20.86)
4–7	9.27 (7.99–10.74)	7.98 (6.36–10.02)	10.49 (8.64–12.74)
8–11	7.16 (6.22–8.24)	5.77 (4.63–7.19)	8.58 (7.14–10.30)
12+	5.20 (4.28–6.31) (*p*<0.001)	4.12 (3.02–5.62) (*p*<0.001)	6.25 (4.87–8.01) (*p* = 0.002)
**Currently employed**			
No	8.08 (7.37–8.85)	6.80 (5.91–7.83)	9.35 (8.29–10.54)
Yes	6.92 (5.81–8.24) (*p* = 0.088)	5.60 (4.23–7.41) (*p* = 0.166)	8.14 (6.51–10.18) (*p* = 0.262)
**Household electricity**			
No	9.09 (5.06–10.25)	7.82 (6.50–9.40)	10.34 (8.82–12.12)
Yes	6.98 (6.25–7.78) (*p*<0.001)	5.71 (4.81–6.78) (*p* = 0.004)	8.21 (7.12–9.46) (*p* = 0.017)
**Household piped water**			
No	8.71 (7.86–9.65)	7.79 (6.68–9.09)	9.61 (8.37–11.02)
Yes	6.59 (5.77–7.51) (*p*<0.001)	4.91 (3.95–6.09) (*p* = 0.001)	8.26 (6.99–9.75) (*p* = 0.091)
**Currently married/cohabiting**			
No	5.49 (4.65–6.47)	4.73 (3.68–6.08)	6.25 (5.02–7.78)
Yes	8.99 (8.18–9.87) (*p*<0.001)	7.41 (6.40–8.59) (*p*<0.001)	10.51 (9.31–11.86) (*p* = 0.002)
**Parity**			
Primiparous	7.62 (6.38–9.09)	5.66 (4.31–7.42)	10.21 (8.09–12.88)
Parity ≥2	7.85 (7.17–8.60) (p = 0.774)	6.79 (5.89–7.82) (p = 0.239)	8.80 (7.12–9.91) (p = 0.269)
**Travel time to facility (min)**			
<30	7.00 (6.08–8.07)	5.86 (4.73–7.26)	8.26 (6.83–9.98)
30–60	8.25 (7.36–9.24)	6.85 (5.73–8.19)	9.57 (8.26–11.09)
60+	8.12 (6.67–9.88) (*p* = 0.290)	7.15 (5.24–9.74) (*p* = 0.462)	8.92 (6.93–11.49) (*p* = 0.499)
**WHO stage at enrollment**			
1	7.60 (6.85–8.42)	6.32 (5.46–7.31)	9.56 (8.25–11.08)
2	8.74 (7.41–10.30)	7.87 (5.98–10.36)	9.31 (7.58–11.43)
3	7.13 (5.72–8.89)	4.92 (2.79–8.66)	7.75 (6.10–9.85)
4	7.58 (4.08–14.09) (*p* = 0.491)	0 (––) (*p*<0.001)	8.18 (4.40–15.20) (*p* = 0.458)
**CD4 at enrollment (cells/µl)**			
≥500	8.21 (7.19–9.37)	7.34 (6.29–8.56)	12.39 (9.56–16.06)
350–499	7.52 (6.24–9.07)	5.88 (4.55–7.61)	10.91 (8.31–14.31)
20–349	7.23 (6.06–8.63)	4.32 (2.85–6.57)	8.47 (6.97–10.30)
<200	7.87 (6.66–9.31) (*p* = 0.620)	4.09 (1.02–16.37) (0.113)	7.98 (6.74–9.45) (*p* = 0.033)
**Male partner enrolled**			
No	6.75 (6.10–7.46)	5.82 (5.00–6.76)	7.74 (6.76–8.85)
Yes	10.81 (9.44–12.38) (*p*<0.001)	8.88 (7.08–11.14) (*p* = 0.005)	12.32 (10.39–14.60) (*p*<0.001)
***Follow–up characteristics***			
**Contraceptive use**			
None	14.38 (12.88–16.05)	13.12 (11.13–15.46)	15.61 (13.45–18.12)
Any barrier/nonbarrier method	5.08 (4.51–5.72) (*p*<0.001)	3.80 (3.12–4.61) (*p*<0.001)	6.34 (5.46–7.36) (*p*<0.001)
Condoms	7.33 (6.38–8.42)	5.70 (4.55–7.14)	8.89 (7.45–10.61)
Any nonbarrier method	2.59 (2.10–3.19) (*p*<0.001)	1.84 (1.30–2.60) (*p*<0.001)	3.36 (2.60–4.36) (*p*<0.001)
Injectable methods	1.53 (1.09–2.15)	1.10 (0.63–1.94)	1.97 (1.28–3.01)
Oral contraception	4.06 (2.56–6.44)	3.11 (1.55–6.21)	5.38 (2.89–10.00)
All hormonal methods	1.96 (1.49–2.58)	1.49 (0.96–2.30)	2.47 (1.74–3.51)
IUD use	0	0	0
Other^a^	5.22 (3.81–7.14)	3.22 (1.83–5.67)	7.20 (4.94–10.50)
**Most recent CD4 count (cells/µl)**			
≥500	7.60 (6.64–8.69)	7.23 (6.10–8.55)	14.51 (11.59–18.17)
350–499	6.86 (5.84–8.06)	6.74 (5.37–8.45)	9.27 (7.37–11.66)
200–349	6.04 (5.11–7.14)	5.00 (3.53–7.07)	8.05 (6.65–9.75)
<200	5.71 (4.50–7.24) (*p* = 0.069)	1.23 (0.17–8.74) (*p* = 0.061)	7.56 (5.94–9.60) (*p* = 0.002)

a Other contraception includes natural family planning, diaphragm use, and traditional methods of family planning.

IUD, intrauterine device.

Women who reported any contraceptive use during follow-up had lower pregnancy rates than those who did not. This association was due largely to the effect of nonbarrier methods, particularly injectable hormonal contraceptive use (unadjusted RR during both the pre-ART and on-ART periods compared to no contraceptive use, 0.18; 95% CI 0.13–0.24). Condom use was associated with a higher pregnancy rate compared to nonbarrier method use, but the rate was still lower than the rate among women reporting no contraception. Overall, 89 pregnancies (15%) occurred while women reported using a nonbarrier method, and 201 while women reported using condoms (34%).

Lower current CD4 counts during follow-up were associated with lower pregnancy rates in both the pre-ART and on-ART periods. Compared to women with current CD4 counts <200 cells/µl, women with current CD4 counts ≥500 cells/µl had more than five times the rate of pregnancy during the pre-ART period (crude RR, 5.87; 95% CI 1.04–233.62); this association was smaller but still significantly higher during the on-ART period (crude RR, 1.92; 95% CI 1.36–2.70).

### Multivariate Analysis

**Figure 3:**
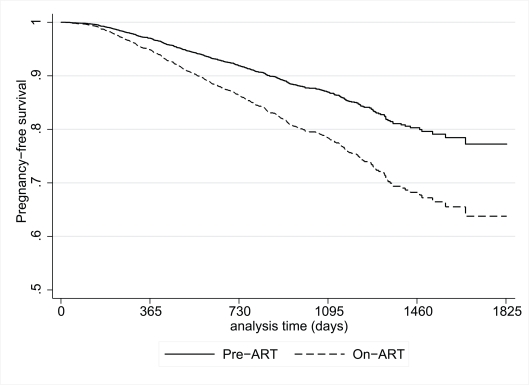
Plot of the postestimation pregnancy-free survival function from a proportional hazards model for participants during the pre-ART and on-ART periods. Model adjusted for participant age, education, nonbarrier contraceptive use, marital/cohabiting status, parity, enrollment of partner into the program, and current CD4 count.

In a model examining pregnancy during the overall observation period (combining pre-ART and on-ART periods) adjusted for participant demographic and clinical characteristics, use of ART was associated with an almost 80% increased risk of pregnancy (HR, 1.74; 95% CI 1.19–2.54) (Table 3). In the same model, the cumulative risk of pregnancy after 4 y of follow-up among women in the pre-ART group was less than 20%, compared to 33% among women using ART (Figure 3). The association between ART use and pregnancy persisted when the analysis was restricted to women who initiated ART during follow-up (HR, 1.81; 95% CI 1.00–3.26). Other characteristics that were significantly associated with an increased risk of pregnancy in the overall model included younger age, lower levels of education, being married or cohabiting, having a male partner enrolled into the program, nonuse of nonbarrier forms of contraception, and higher current CD4 cell counts. When the model was restricted to the pre-ART and on-ART periods many of these associations persisted (Table 3).

**Table 3:** Cox's proportional hazards models examining the association between ART and incident pregnancy, presented as adjusted HRs with 95% CIs.

Characteristics	Overall (*n* = 4,531)		Pre–ART (*n* = 3,951)		On-ART (*n* = 1,980)	
	HR	95% CI	HR	95% CI	HR	95% CI
**On-ART (versus pre-ART)**	1.74	1.19–2.54	—	—	—	—
**Age (continuous)**	0.93	0.91–0.94	0.93	0.90–0.97	0.92	0.91–0.94
**Years (y) of education**						
0–3	1.0	—	1.0	—	1.0	—
4–7	0.84	0.65–1.08	0.76	0.50–1.15	0.95	0.73–1.24
8–11	0.70	0.56–0.89	0.61	0.46–0.81	0.81	0.62–1.06
12+	0.52	0.41–0.67	0.47	0.30–0.75	0.59	0.45–0.77
**Married or cohabiting (versus not)**	1.40	1.18–1.65	1.36	1.12–1.65	1.45	1.08–1.97
**Parity ≥2 (versus primiparous)**	1.17	0.96–1.44	1.37	0.90–2.08	1.02	0.72–1.45
**Partner enrolled into program (versus not)**	1.38	1.11–1.72	1.11	0.61–2.07	1.64	1.09–2.45
**Use of nonbarrier contraception (versus condoms only or no method)**	0.31	0.19–0.50	0.22	0.12–0.41	0.42	0.24–0.72
**Baseline WHO stage**						
1	—	—	1.0	—	—	—
2	—	—	1.05	0.79–1.40	—	—
3 & 4	—	—	0.75	0.37–1.52	—	—
**Most recent CD4 count (cells/µl)**						
≥500	1.0	—	1.0	—	1.0	—
350–499	0.83	0.67–1.02	0.87	0.70–1.07	0.73	0.51–1.05
200–349	0.68	0.44–1.05	0.58	0.44–0.77	0.68	0.35–1.33
<200	0.68	0.41–1.14	0.17	0.05–0.59	0.70	0.34–1.47

Each model is adjusted for all covariates listed.

In separate proportional hazards models including the overall observation period stratified by country and adjusted for all covariates shown in Table 3, the association between ART use and incidence of pregnancy remained relatively constant across countries although not all associations achieved statistical significance. The weakest association was observed in Rwanda (HR, 1.07; 95% CI 0.28–4.01), followed by Uganda (HR, 1.33; 95% CI 0.59–2.98), and Cote d'Ivoire (HR, 1.35; 95% CI 0.49–3.70); stronger associations were observed at the sites in Kenya (HR, 1.94; 95% CI 0.84–4.48), South Africa (HR, 2.33; 95% CI 0.70–7.73), and Zambia (HR, 2.82; 95% CI 0.80–9.97).

## Discussion

The incidence of pregnancy among women enrolled in HIV care and treatment programs in sub-Saharan Africa has important implications for the health of women and their infants. Our findings indicate a high overall incidence of pregnancy (more than seven pregnancies per 100 woman-years of observation) and a significant association between the use of ART and increased incidence of pregnancy (adjusted HR, 1.74). Within 4 y of follow-up, one-third of women who initiated ART experienced a pregnancy, highlighting the urgent need to make pregnancy-related services a central component of HIV care and ART programs.

A series of biological and behavioral factors may influence the association between the use of ART and increased incidence of pregnancy. It is possible that the rapid improvements in health and quality of life that take place with ART initiation lead to increased sexual activity, particularly for those with stable partnerships [20]. Improving health with ART use may contribute to increased fertility desires through psychological mechanisms of increased hopefulness about the future and improved mental health, as well as through increases in sexual activity and new partner acquisition [21]. Related to this we found that more advanced HIV disease (as indicated by higher WHO staging and lower CD4 cell counts) were strongly associated with reduced incidence of pregnancy, consistent with previous findings [22],[23]. In addition it is important to note that approximately 30% of pregnancies in sub-Saharan Africa are unintentional [24],[25], regardless of the HIV status of the mother, and in this light other factors may also play a role. In particular, it is plausible that improvements in immunological functioning with ART increase female fecundity compared to pre-ART levels, though the mechanisms through which ART use may reduce pregnancy loss are not well understood.

We do not have data on sexual activity or fertility intentions to help elucidate the reasons why ART use may increase fertility. There is evidence that fertility intentions change over the natural history of HIV infection. In keeping with the findings of qualitative studies, a recent analysis of HIV-infected and -uninfected women from Malawi [26] suggests that fertility intentions are diminished by a diagnosis with HIV/AIDS and this may continue during the pre-ART period. In contrast, both fertility intentions and sexual activity may increase after ART initiation, as suggested by a cohort of Ugandan adults and a cross-sectional study from South Africa [27],[28]. Importantly, these and other studies [9],[29] also suggest that men are likely to express greater fertility desires than women. In this study, we did not have data on male partners' fertility desires. However we observed an increase in the rate of pregnancy in women who were married or cohabiting, as well as those who had a male partner enrolled into the program, and most male partners enrolled into the program were receiving ART. These associations suggest the important role that partners are likely to play in fertility among HIV-infected women, both in shaping fertility-related decision making and providing an opportunity to conceive [30].

Nonbarrier contraceptives, although used by only approximately one-fifth of women, were strongly associated with a reduced incidence of pregnancy. This reduction was driven chiefly by use of injectable hormonal contraceptives and the intrauterine device (IUD), underscoring the valuable role of these methods for pregnancy prevention. A substantial proportion of pregnancies in this cohort occurred during self-reported method use, suggesting that method failure, noncompliance, or over-reporting of method use may occur in many instances. Among women reporting any contraceptive method, the highest pregnancy incidence was observed among those using condoms, highlighting the limited effectiveness of condoms as a stand-alone contraceptive method and emphasizing the importance of dual method use. The association between oral contraceptive use and higher pregnancy rates compared to other nonbarrier methods may reflect nonadherence with oral contraceptive pills or drug interactions between antiretroviral and oral contraceptive drugs [31]. There is preliminary evidence that nevirapine through its inducer effect on hepatic P450 enzymes is associated with decreased blood levels of combined oral contraceptives [32].

The high incidence of pregnancy, coupled with the low prevalence of contraceptive use, underscore the importance of addressing fertility-related issues within HIV care and treatment programs in sub-Saharan Africa. The increasing rate of new pregnancies we observed with duration of follow-up on-ART is consistent with findings from prior studies [27],[28]. However, the design and operation of most HIV treatment services do not explicitly acknowledge the likelihood or the actual occurrence of pregnancy [3]. Yet new pregnancies among HIV-infected women enrolled in such programs have major implications for the clinical management of the woman's health in addition to requiring attention for effective PMTCT [4]. HIV care and treatment services must strengthen medical as well as psychosocial care to address fertility desires and plans for both women and men with HIV infection. For women who do not wish to become pregnant, making effective methods of contraception as well as safe abortion services available is critical. For women who desire a child, appropriate planning prior to pregnancy, the choice of antiretroviral drugs, and the administration of effective PMTCT interventions will optimize outcomes for both the mother and child [16].

The data presented in this paper were derived from systematic, standardized data collection in an HIV care and treatment program across seven countries in sub-Saharan Africa. The clinical sites that participated in this initiative are broadly representative of public sector HIV care and ART delivery programs, and thus the findings are likely to be more generalizable than evidence from small research cohorts [10],[33]. We observed that the rates of incident pregnancy among HIV-infected women range 5-fold across countries, which may reflect variation in patient demographics and/or access to contraception and related counseling. There was no specific package of fertility-related counseling and services provided as part of the MTCT-Plus Initiative, and site-specific heterogeneity in promotion of family planning services may contribute to the observed differences. Despite this, the association between ART use and pregnancy was surprisingly consistent across countries; in a separate sensitivity analysis (unpublished data), this association was minimally altered by the removal of any single country from the analysis.

A distinctive strength of these data is that the analyses included women followed up for incident pregnancies both before and after ART initiation, as sites enrolled women into ongoing HIV care regardless of disease stage or ART eligibility. However, our findings should be interpreted in light of several limitations. Although data were collected on standardized forms, the ascertainment of new pregnancies was based on patient self-report and clinical assessment rather than laboratory assays, a factor that may have led to the underdetection of pregnancies. A proportion of pregnancies are likely to be lost prior to clinical detection, which may occur more frequently in HIV-infected women [34], making these estimates of pregnancy incidence conservative. However, it is unlikely that this ascertainment issue would have been influenced by women's use of ART. Similarly, imprecision in the clinical dating of incident pregnancies and resultant inaccuracy in the estimation of conception dates is also unlikely to have been influenced by ART status (or other participant characteristics). It is also important to note that this cohort was comprised of parous women; although pregnancy rates are likely to be higher among nulliparous women, it is unclear whether the impact of ART use and other covariates would differ in women without children.

In summary, our findings indicate that the incidence of pregnancy increases significantly after the initiation of ART. Although the precise reasons for this increase require additional research, HIV care and treatment programs have an important opportunity to address women's fertility intentions and to shape their services to address the needs of the women and their families over time.

## Abbreviations

ART: antiretroviral therapy
CI: confidence interval
HR: hazard ratio
IQR: interquartile range
PMTCT: prevention of mother-to-child transmission
PY: person-year
RR: rate ratio
